# Targeted Serum Organic-Acid Profiling Identifies Candidate Metabolic Signatures in Peripartum Cardiomyopathy: A Case-Control Study

**DOI:** 10.3390/ijms27146451

**Published:** 2026-07-20

**Authors:** Yasemin Behram Kandemir, Ersin Doğanözü, Ünal Güntekin, Veysel Tosun

**Affiliations:** 1 Department of Anatomy, Faculty of Medicine, Istanbul Aydın University, 34295 Istanbul, Turkey; ybehramkandemir@gmail.com; 2 Department of Cardiology, Faculty of Medicine, Akdeniz University, 07070 Antalya, Turkey; unalguntekin@akdeniz.edu.tr; 3 Department of Cardiology, Mehmet Akif Inan Training and Research Hospital, University of Health Sciences, 63200 Sanliurfa, Turkey; veyseltosun@sbu.edu.tr

**Keywords:** peripartum cardiomyopathy, organic acids, targeted metabolomics, biomarkers, receiver operating characteristic analysis

## Abstract

Peripartum cardiomyopathy (PPCM) is a potentially life-threatening form of heart failure that occurs toward the end of pregnancy or in the months following delivery. Biomarkers that may support the recognition and characterization of PPCM remain limited. We investigated whether targeted serum organic-acid profiling could identify candidate metabolites associated with PPCM. In this case–control study, serum samples from women with PPCM (*n* = 40) and healthy controls (*n* = 40) were analyzed using a targeted organic-acid panel. Log_2_-transformed metabolite abundances were compared using Welch’s *t*-test, with Benjamini–Hochberg false discovery rate (FDR) correction correction and Hedges’ g effect sizes. Distributional assumptions were assessed using group-specific Shapiro–Wilk tests, and Mann–Whitney U tests were performed as a non-parametric sensitivity analysis. Homogentisic acid was increased in PPCM (*log*_2_ *fold change (log*_2_*FC)* = 0.696; *FDR q* = 9.8 × 10^−6^), whereas lactic acid was decreased (*log*_2_*FC* = −0.701; *FDR q* = 0.00773). Both findings remained significant in the Mann–Whitney U sensitivity analysis (*BH-FDR q* = 5.63 × 10^−5^ and q = 0.0114, respectively). The best-performing single metabolite showed an area under the receiver operating characteristic curve(AUC) of 0.818 (*95% CI, 0.723–0.899*). The multivariable model achieved a cross-validated AUC of 0.708 (*95% CI, 0.590–0.813*), with a sensitivity of 0.800 and a specificity of 0.550. Targeted organic-acid profiling revealed a distinct metabolic signal in PPCM, highlighted by higher homogentisic acid and lower lactic acid levels. These exploratory findings warrant confirmation in larger prospective studies with external validation.

## 1. Introduction

Peripartum cardiomyopathy (PPCM) is an uncommon but potentially life-threatening cause of heart failure with reduced ejection fraction that presents in previously healthy women toward the end of pregnancy or within the months following delivery, after exclusion of other identifiable causes of cardiomyopathy [1,2,3]. Despite advances in heart failure therapy, PPCM continues to contribute substantially to maternal morbidity and mortality worldwide, with marked geographic and ethnic variation in incidence and outcomes [2,3,4]. Clinical recognition can be challenging because early symptoms overlap with normal pregnancy and postpartum physiology, often resulting in delayed diagnosis and preventable complications [3,4]. Contemporary position statements emphasise the need for improved risk stratification tools and clinically actionable biomarkers to support timely diagnosis and individualised management [1,2].

The pathophysiology of PPCM is multifactorial and incompletely understood. Current concepts highlight a convergence of peripartum hemodynamic load with susceptibility factors that promote myocardial injury, inflammation, and maladaptive remodelling [1,2,3,4]. A major mechanistic framework involves oxidative stress–dependent cleavage of prolactin into a 16-kilodalton (kDa) fragment with anti-angiogenic and pro-apoptotic properties, coupled with downstream signalling mediators such as microRNA-146a (miR-146a) [5,6,7]. In parallel, cardiac angiogenic imbalance has been implicated as a driver of PPCM, providing a biologically coherent link between pregnancy-related vascular signalling and myocardial dysfunction [6]. These insights have also motivated disease-specific therapeutic strategies (e.g., bromocriptine added to guideline-based heart failure therapy in selected patients), underscoring the translational relevance of mechanistic pathways in PPCM [8].

A growing body of evidence suggests that metabolic remodelling is not merely a consequence of heart failure, but can contribute to disease initiation and progression. Metabolomics offers an unbiased framework to quantify metabolic intermediates across pathways central to myocardial energy production, redox balance, and mitochondrial function, including organic acids, amino acids, and related intermediates [9]. In heart failure populations, targeted metabolomic profiling has identified circulating metabolite signatures associated with clinical severity and outcomes, supporting the potential of metabolomics for biomarker discovery and risk stratification [10,11]. Importantly, targeted assays can reliably quantify metabolite classes directly linked to cardiac energy metabolism, such as tricarboxylic acid (TCA) cycle intermediates, glycolytic/pyruvate metabolites, and other organic acids, enabling clinically interpretable readouts of systemic and myocardial metabolic status [12].

In PPCM specifically, “omics” studies have predominantly focused on hormonal and vascular mediators and on non-metabolite biomarkers, including microRNA-based signatures and proteomic profiling [7,13]. Only recently have downstream myocardial proteomic and metabolomic changes been characterised in PPCM tissue, suggesting distinct molecular features related to cellular and metabolic remodelling [14]. However, clinically interpretable targeted metabolite panels—particularly those centred on organic acids reflecting core mitochondrial and intermediary metabolism—remain underexplored in PPCM. Therefore, in this case–control study including women with PPCM (*n* = 40) and healthy controls (*n* = 40), we applied targeted serum organic-acid profiling using a CE-marked in vitro diagnostic (CE-IVD) liquid chromatography–tandem mass spectrometry (LC–MS/MS) platform to identify candidate metabolites associated with PPCM, estimate effect sizes with correction for multiple testing, assess discriminatory performance by receiver operating characteristic (ROC) analysis with internal cross-validation, and explore pathway-level signals within the measured metabolite panel.

## 2. Results

### 2.1. Baseline Clinical Characteristics of the Study Population

Baseline clinical characteristics of the study population are summarized in Table 1. Age, postpartum interval, body mass index, and selected obstetric variables were broadly comparable between the PPCM and control groups. As expected, left ventricular systolic function was impaired in the PPCM group. Routine laboratory variables, including inflammatory markers, renal function parameters, and liver enzymes, were also assessed to support clinical interpretation of the metabolomic findings. N-terminal pro-B-type natriuretic peptide (NT-proBNP)measurements were available for the PPCM group as part of standard clinical evaluation.

The postpartum interval at blood sampling was comparable between the PPCM and control groups [36 days (IQR 20–63) vs. 34 days (IQR 19–58), respectively; *p* = 0.61], supporting group-level comparability with respect to postpartum timing.

### 2.2. Differential Organic-Acid Profile in PPCM Versus Controls

**Figure 1:**
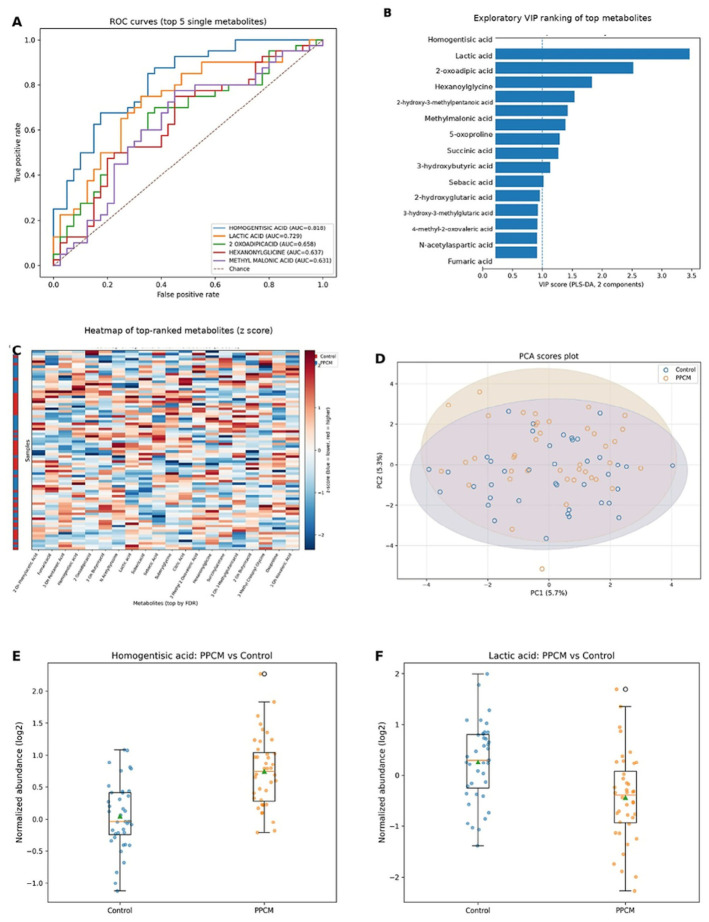
Integrated serum organic-acid profiling in peripartum cardiomyopathy (PPCM) and controls. (**A**) Receiver operating characteristic (ROC) curves of the top five single metabolites; each colored curve represents an individual metabolite, as indicated in the legend, and the diagonal gray line represents the no-discrimination reference line. (**B**) Exploratory variable importance in projection (VIP) ranking of top metabolites derived from partial least squares discriminant analysis (PLS-DA) using two components; bar length indicates the VIP score. (**C**) Heatmap of top-ranked metabolites shown as z scores across samples; red indicates relatively higher standardized abundance and blue indicates relatively lower standardized abundance. (**D**) Principal component analysis (PCA) scores plot based on the first two principal components; each point represents an individual participant, colours indicate study groups, and shaded ellipses indicate group-level dispersion. (**E**) Group-wise distribution of homogentisic acid. (**F**) Group-wise distribution of lactic acid. In panels (**D**–**F**), blue represents controls and orange represents PPCM. Note: PLS-DA/VIP findings are presented as exploratory feature-ranking results.

Targeted serum organic-acid profiling identified a limited number of robust between-group differences after correction for multiple testing. Homogentisic acid was significantly higher in the PPCM group than in controls (0.74 ± 0.56 vs. 0.05 ± 0.53, log_2_ fold change *(log*_2_*FC*) = 0.696, fold change *(FC)* = 1.620; Welch’s *p* = 1.82 × 10^−7^; false discovery rate *(FDR)* q = 9.8 × 10^−6^; *Hedges’ g* = 1.269), whereas lactic acid was significantly lower in PPCM (−0.44 ± 0.87 vs. 0.26 ± 0.77, *log*_2_*FC* = −0.701, *FC* = 0.615; *Welch’s p* = 2.86 × 10^−4^; *FDR q* = 0.00773; *Hedges’ g* = −0.842) (Table 2; Figure 1A,B). Several additional metabolites, including 2-oxoadipic acid and hexanoylglycine, showed nominal between-group differences but did not remain significant after FDR adjustment (Table 2).

The distributional characteristics of the analyzed log_2_-transformed metabolites were assessed separately in the PPCM and healthy control (HC) groups using the Shapiro–Wilk normality test, and the results are summarized in Supplementary Table S1. As a non-parametric sensitivity analysis, between-group differences were also evaluated using two-sided Mann–Whitney U tests, followed by Benjamini–Hochberg FDR correction. The two main FDR-significant findings from the primary Welch analysis remained consistent in the Mann–Whitney sensitivity analysis: homogentisic acid was higher in PPCM (Mann–Whitney *p* = 1.04 × 10^−6^; BH-FDR q = 5.63 × 10^−5^), and lactic acid was lower in PPCM (Mann–Whitney *p* = 4.21 × 10^−4^; BH-FDR q = 0.0114). These results support the robustness of the main findings to a non-parametric analytical approach (Supplementary Table S1).

### 2.3. Discriminatory Performance of Candidate Metabolites

Single-metabolite ROC analyses identified homogentisic acid as the best-performing individual discriminator (AUC = 0.818), followed by lactic acid (AUC = 0.729), whereas the remaining top-ranked metabolites showed comparatively lower AUC values (Figure 1C). In exploratory multivariable classification, a logistic regression model evaluated by internal 5-fold cross-validation yielded an AUC of 0.708 (95% CI 0.590–0.813). At the Youden-optimal threshold (0.211), sensitivity was 0.800 and specificity was 0.550 (Table 3). Given the modest sample size and lack of external validation, these multivariable results should be regarded as internally validated, hypothesis-generating findings rather than as evidence of a clinically deployable prediction model.

### 2.4. Multivariate Structure and Metabolite Ranking

Unsupervised multivariate exploration demonstrated modest separation between PPCM and controls on principal component analysis (PCA) (Figure 1F). Consistently, a heatmap of the top differential metabolites ranked by FDR showed partial group-level patterning, with the strongest contributions arising from the highest-ranked features (Figure 1E). In partial least squares discriminant analysis (PLS-DA), variable importance in projection (VIP) ranking identified homogentisic acid (VIP = 3.464) and lactic acid (VIP = 2.520) as the most influential metabolites, followed by 2-oxoadipic acid (VIP = 1.829) and hexanoylglycine (VIP = 1.537), among others (Table 4; Figure 1D). Because formal PLS-DA validation metrics, such as R^2^/Q^2^ estimates, permutation testing, or cross-validated analysis of variance (CV-ANOVA), were not generated, these supervised rankings should be interpreted as exploratory feature-prioritization results rather than evidence of a robust validated class-separation model.

### 2.5. Exploratory Pathway Signals Within the Measured Panel

Within-panel over-representation analysis (ORA) suggested glycolysis/pyruvate metabolism as the highest-ranking pathway-level signal (k/K = 1/2; enrichment ratio = 13.5; *p* = 0.0734; FDR = 0.66), but no pathway remained significant after FDR correction (Table 5). These findings should therefore be considered exploratory and hypothesis-generating, particularly given the small number of metabolites that remained significant after multiple-testing adjustment.

## 3. Discussion

In this targeted serum organic-acid study, we identified a compact set of candidate metabolic features associated with peripartum cardiomyopathy (PPCM). The principal findings were a marked increase in homogentisic acid and a significant reduction in lactic acid in PPCM compared with healthy controls. These metabolites were consistently prioritized across complementary analytical approaches, including FDR-based ranking, ROC analysis, and exploratory VIP ranking, suggesting that PPCM in this cohort may be characterized by a focused pattern of organic-acid perturbation rather than by broad separation across the entire measured panel.

### 3.1. Interpretation in the Context of PPCM Pathophysiology

PPCM is a heterogeneous syndrome in which late-gestational and postpartum physiological stressors interact with underlying susceptibility factors to precipitate myocardial dysfunction. Contemporary position statements and state-of-the-art reviews suggest that PPCM likely reflects converging mechanisms involving vascular and hormonal perturbations, oxidative stress, inflammation, and myocardial remodeling, while also underscoring the unmet need for biomarkers that may facilitate earlier recognition and improved risk stratification [1,2,3,15].

Mechanistic studies have linked PPCM to oxidative stress-dependent cleavage of prolactin into a 16-kDa fragment with downstream anti-angiogenic and pro-apoptotic effects [5]. In parallel, systemic angiogenic imbalance has emerged as a plausible contributor to PPCM susceptibility, particularly in the setting of hypertensive disorders of pregnancy and multiple gestation, supporting a vascular–cardiac axis in disease pathogenesis [6]. In addition, *miR-146a* has been proposed as a mediator and candidate biomarker associated with prolactin-pathway activation and endothelial dysfunction in PPCM [7]. Against this background, the metabolic alterations observed in our organic-acid profile may represent downstream consequences of myocardial stress, endothelial dysfunction, and altered substrate handling during the peripartum period [1,5,6,7].

### 3.2. Homogentisic Acid and Aromatic Amino-Acid/Tyrosine Catabolism

Homogentisic acid is an intermediate of phenylalanine and tyrosine metabolism. In the present study, it was the strongest individual metabolite associated with PPCM. However, because of the cross-sectional observational design, this finding should not be interpreted as evidence that homogentisic acid has a causal role in PPCM pathogenesis. The observed difference may reflect broader alterations in amino-acid catabolism, redox balance, renal or hepatic handling, diet, microbiome-related metabolism, medication exposure, or postpartum physiological changes. Therefore, homogentisic acid should be considered a candidate metabolite for future validation rather than a mechanistic mediator identified by the present study [16].

### 3.3. Lactic Acid and Myocardial Energy Metabolism

Lactate is closely related to pyruvate metabolism, redox balance, and systemic substrate handling [17]. In the present cohort, serum lactic acid was lower in PPCM than in controls. However, this cross-sectional finding cannot determine whether lower lactate is a cause, consequence, or nonspecific correlate of PPCM. The observed difference may be influenced by postpartum timing, systemic metabolic adaptation, tissue uptake and clearance, hemodynamic status, renal or hepatic function, diet, and medication exposure. Therefore, lactate-related findings should be interpreted as preliminary and hypothesis-generating. Longitudinal studies are needed to determine whether lactate changes are related to disease severity, recovery, or treatment response.

### 3.4. Clinical Meaning: Discrimination Performance and Potential Utility

The present study was not designed to establish a clinical screening, diagnostic, or prognostic test for PPCM. Rather, it aimed to identify candidate organic-acid signals associated with PPCM for future validation. In our dataset, homogentisic acid showed the strongest single-metabolite discrimination, whereas the multivariable model showed only modest internally cross-validated performance. Therefore, these metabolites should not be considered stand-alone diagnostic biomarkers at this stage.

The potential clinical application scenario remains undefined [17,18,19]. In particular, the present data do not establish whether these metabolites are useful for early screening, differential diagnosis, prognostic assessment, or monitoring of recovery [15]. NT-proBNP was available only for the PPCM group as part of routine clinical evaluation, and *miR-146a* was not measured in this cohort. Therefore, we could not perform a head-to-head comparison or incremental diagnostic analysis combining organic-acid metabolites with established or previously proposed PPCM biomarkers. Future prospective studies should evaluate whether candidate metabolites add value beyond NT-proBNP, echocardiographic parameters, *miR-146a*, and clinical risk factors.

### 3.5. Methodological Considerations: Supervised Models and Pathway Analysis

Supervised approaches such as PLS-DA/VIP may be useful for exploratory feature prioritization, but they remain vulnerable to optimistic interpretation when validation is limited. Methodological work in metabolomics has emphasized the importance of rigorous cross-validation and cautioned against overinterpretation of score plots and variable rankings [20,21]. In the present study, although discrimination was assessed using internal cross-validation, formal PLS-DA validation metrics, including R^2^/Q^2^ estimates, permutation testing, and CV-ANOVA, were not generated, and no external validation cohort was available. Accordingly, the PLS-DA/VIP findings should be regarded as supportive and hypothesis-generating rather than as evidence of a robust validated separation model.

Pathway-level interpretation also warrants caution, as enrichment results are sensitive to study design, background definitions, metabolite mapping, and significance thresholds [22,23]. Although glycolysis/pyruvate metabolism emerged as the top-ranked pathway-level signal, no pathway remained significant after FDR correction and the degree of overlap was limited. These findings should therefore be interpreted as exploratory and hypothesis-generating rather than as definitive evidence of pathway dysregulation in PPCM. More comprehensive pathway-level analyses in broader metabolomic datasets, together with external validation, will be important for establishing the biological and clinical relevance of these observations [24].

### 3.6. Strengths, Limitations, and Future Directions

Strengths of the present study include the use of a standardized targeted LC–MS/MS workflow, enabling clinically interpretable quantification of organic acids while reducing analytical variability. Several limitations should also be acknowledged. First, the case–control and cross-sectional design does not allow causal inference or assessment of temporal metabolite changes during PPCM development and recovery. Second, the modest sample size and single-center design limit statistical power and generalizability, particularly for smaller metabolite differences, pathway-level analyses, and multivariable classification. Third, although postpartum timing was comparable between groups at the group level, the study did not use strict individual one-to-one matching by postpartum day, and the sampling window remained relatively broad. Therefore, residual confounding related to postpartum physiological changes cannot be fully excluded. Fourth, dietary habits, gut microbiota composition, medication exposure, renal and hepatic function, and common gestational disorders such as preeclampsia and gestational diabetes mellitus were not fully controlled in the present analysis. These factors may influence systemic organic-acid levels and limit extrapolation of the findings. Finally, NT-proBNP was not available in both groups and *miR-146a* was not measured; therefore, the incremental value of the identified metabolites beyond established or proposed PPCM biomarkers could not be evaluated. Future work should prioritize larger multicenter cohorts, longitudinal sampling linked to recovery and adverse outcomes, and integrative models combining metabolites with clinical covariates, echocardiographic data, NT-proBNP, and miRNA-based biomarkers [1,16,17,22,23,24].

In summary, targeted serum organic-acid profiling identified a compact set of candidate metabolic features associated with PPCM, driven primarily by increased homogentisic acid and decreased lactic acid. These findings should be interpreted as exploratory associations rather than evidence of causal involvement or immediate clinical applicability. Further prospective studies are required to validate these candidate metabolites, clarify their temporal behavior, and determine whether they provide incremental value beyond established clinical and echocardiographic assessment.

## 4. Materials and Methods

### 4.1. Study Design and Population

This study was designed as a case–control investigation comparing patients with peripartum cardiomyopathy (PPCM) and healthy controls. A total of 80 participants were included (PPCM, *n* = 40; controls, *n* = 40). PPCM was defined as new-onset heart failure secondary to left ventricular systolic dysfunction presenting toward the end of pregnancy or within 5 months postpartum, in the absence of another identifiable cause of cardiomyopathy. All PPCM patients had left ventricular ejection fraction (LVEF) < 45% on transthoracic echocardiography at diagnosis. Alternative etiologies were excluded by clinical assessment and standard diagnostic work-up; ischemic heart disease was excluded based on history, electrocardiography, and cardiac biomarker evaluation as indicated, and, when clinically warranted, non-invasive imaging and/or invasive coronary angiography according to routine practice.

Controls were recruited from women without symptoms or signs of heart failure, without known structural heart disease, and without clinical evidence of cardiovascular disease. Controls were recruited during the same study period and from the same catchment area and were selected to be broadly comparable to the PPCM group with respect to age and postpartum status whenever feasible.

Exclusion criteria applied to both groups included acute infectious or inflammatory conditions at sampling, active malignancy, severe hepatic failure, end-stage renal disease, and inadequate sample quality (e.g., marked hemolysis or severe lipemia).

All participants included in the present analysis were sampled during the postpartum period. Controls were recruited during the same study period and from the same catchment area as PPCM cases. Eligible controls were selected to achieve broad comparability with the PPCM group at the group level with respect to age and postpartum timing. Although individual one-to-one matching by postpartum day was not performed, the distributions of postpartum interval at sampling were statistically comparable between groups, as reported in Table 1.

The study was conducted at a single clinical center. Therefore, the study population may not fully represent the clinical and demographic heterogeneity of PPCM across different institutions or geographic regions.

### 4.2. Clinical Characteristics and Laboratory Variables

Baseline clinical characterization included demographic features, postpartum interval, selected obstetric variables, vital signs, echocardiographic findings, and routine laboratory parameters collected using a standardized data collection form. Transthoracic echocardiography was performed according to routine clinical practice, and key parameters included left ventricular ejection fraction (LVEF) and left ventricular dimensions (e.g., left ventricular end-diastolic diameter (LVEDD) and left ventricular end-systolic diameter (LVESD)). Routine laboratory variables comprised inflammatory markers, renal function indices, liver enzymes, and NT-proBNP, where available.

### 4.3. Ethical Approval

The study protocol was approved by the Harran University Rectorate Clinical Research Ethics Committee (Harran Üniversitesi Klinik Araştırmalar Etik Kurulu) (meeting date: 10 January 2022; session: 01; decision no.: 22/01/03). The official approval document is dated 28 January 2022 (no.: 101490; reference/document no.: E-76244175-050.04.04-101490). The study was conducted in accordance with the principles of the Declaration of Helsinki, and written informed consent was obtained from all participants prior to enrollment.

### 4.4. Blood Sampling and Pre-Analytical Processing

Venous blood samples were collected into serum tubes and allowed to clot. Samples were centrifuged at 4000 rpm (~1800× *g*) for 10 min at +4 °C. Serum was aliquoted into polypropylene tubes, transported under cold-chain conditions, and stored at −80 °C until batch analysis. To minimize pre-analytical variability, aliquots were thawed only once immediately prior to analysis. Blood samples for metabolomic analysis were collected prior to the initiation of disease-specific medical therapy for PPCM.

Pre-analytical interference control. Samples were screened for hemolysis and lipemia prior to LC–MS/MS analysis. Hemolysis was assessed using the automated hemolysis index (H-index) generated by the clinical chemistry analyzer; specimens exceeding the laboratory’s predefined exclusion threshold for moderate-to-severe hemolysis were excluded. Markedly lipemic samples were excluded based on routine laboratory inspection and/or analyzer flags.

### 4.5. Targeted Organic-Acid Profiling by LC–MS/MS (JASEM)

Targeted quantification of 54 organic acids in serum was performed using a CE-IVD-certified JASEM Serum Organic Acids LC–MS/MS Analysis Kit (Sem Laboratuvar Cihazları Paz. San. ve Tic. A.Ş., Istanbul, Turkey) on a Shimadzu LCMS-8045 liquid chromatography–tandem mass spectrometry system (Shimadzu Corporation, Kyoto, Japan). A six-point calibration curve was generated using calibration standards supplied with the kit. The stable isotope-labeled internal standard mixture supplied with the kit included adipic acid-^13^C_6_, methylmalonic acid-^2^H_3_, and sodium lactate-^2^H_3_. A total of 54 targeted analytes were measured using the assay panel. Metabolites included in downstream statistical analyses were required to satisfy predefined analytical quality criteria. Missing values and/or concentrations below the limit of quantification were handled according to the assay-specific preprocessing workflow. Analytical reproducibility was monitored using study-specific pooled quality control (QC) samples, and potential batch drift was assessed across the acquisition sequence.

### 4.6. Sample Preparation

Sample preparation followed the standardized kit workflow (“protein precipitate and inject”). Briefly, 100 µL serum was transferred to a centrifuge tube, 50 µL internal standard mixture supplied with the JASEM kit was added and vortexed for 5 s, followed by the addition of 850 µL Reagent-1 supplied with the JASEM kit, vortexing for 5 s, and centrifugation at 4000 rpm for 5 min. The supernatant was transferred to an HPLC vial and injected for LC–MS/MS analysis.

### 4.7. Chromatography and MS Detection

Chromatographic separation was performed using the manufacturer-recommended organic-acid LC method on a JASEM organic acid column (JASEM/Sem Laboratuvar Cihazları Paz. San. ve Tic. A.Ş., Istanbul, Turkey). The column temperature was maintained at 40 °C, and chromatographic separation was conducted using mobile phase A and B gradient solutions supplied with the JASEM kit at a flow rate of 0.4 mL/min. The analytical workflow consisted of two acquisition panels with a 12 min run time per panel, resulting in a total analysis time of 24 min for Panels 1 and 2. Mass spectrometric detection was performed on a Shimadzu LCMS-8045 triple quadrupole LC–MS/MS system (Shimadzu Corporation, Kyoto, Japan) using electrospray ionization in both negative and positive ion modes and multiple-reaction monitoring (MRM) with kit-defined transitions and acquisition parameters.

### 4.8. Analytical Quality Control and Batch Management

Samples were analyzed in batches, and injection order was randomized with respect to clinical group (PPCM vs. control) to minimize run-order effects. A pooled serum quality control (QC) sample, prepared by combining small aliquots of study specimens, was injected at the beginning and end of each batch and after every 10 study samples to monitor analytical stability and potential signal drift. Calibrators, internal standard mixture, reagents, mobile phases, and blank injections supplied with or recommended for the JASEM Serum Organic Acids LC–MS/MS Analysis Kit (Sem Laboratuvar Cihazları Paz. San. ve Tic. A.Ş., Istanbul, Turkey) were included within each batch. Batch acceptance was determined by calibration performance and QC stability; samples failing predefined quality criteria were reanalyzed when sufficient material was available. Laboratory personnel performing LC–MS/MS analyses were blinded to clinical group assignment.

### 4.9. Assay Performance (Public Technical Specifications)

According to the manufacturer’s publicly available technical specifications for the JASEM Serum Organic Acids LC–MS/MS Analysis Kit (Sem Laboratuvar Cihazları Paz. San. ve Tic. A.Ş., Istanbul, Turkey), analyte-specific limits of quantification (LOQ) span approximately 0.03 to 5.85 mg/L, and reported repeatability, expressed as relative standard deviation (%RSD), spans approximately 0.57% to 13.25% across analytes.

### 4.10. Statistical Analysis

Metabolite abundances were analyzed after log_2_ transformation. Log_2_ fold change (log_2_FC) was calculated as the difference in mean log_2_-transformed abundance between the PPCM and control groups; positive values indicate higher abundance in PPCM, whereas negative values indicate lower abundance. Between-group comparisons were performed using two-sided Welch’s *t*-tests, which were selected because they do not require the assumption of equal variances between groups. Distributional characteristics were assessed separately within the PPCM and healthy control groups using the Shapiro–Wilk test and visual inspection of Q–Q plots. To account for multiple comparisons, *p* values were adjusted using the Benjamini–Hochberg false discovery rate procedure, with BH-FDR q < 0.05 considered statistically significant. Effect sizes were summarized using Hedges’ g. As a non-parametric sensitivity analysis, two-sided Mann–Whitney U tests were performed for each analyzed metabolite. Mann–Whitney U *p* values were adjusted using the Benjamini–Hochberg procedure across all 54 analyzed metabolites. Unadjusted *p* values and BH-FDR q values are reported in Supplementary Table S1 together with the group-specific Shapiro–Wilk *p* values.

Discriminatory performance was evaluated at two levels. First, individual metabolites were assessed using receiver operating characteristic (ROC) analysis. Second, an exploratory multivariable logistic regression classifier was constructed using a small set of top-ranked candidate metabolites prioritized for joint evaluation in the present dataset. Model performance was estimated using stratified 5-fold cross-validation. No additional scaling or preprocessing was applied to the multivariable model. The area under the ROC curve (AUC) was used as the primary discrimination metric, and 95% confidence intervals were obtained by bootstrap resampling of the cross-validated predicted probabilities with 2000 iterations. The operating threshold was selected using the Youden index, and the corresponding sensitivity and specificity were reported as internally validated estimates. Given the modest sample size, case–control design, and lack of external validation, the multivariable classifier was interpreted as exploratory and hypothesis-generating.

For multivariate structure, principal component analysis (PCA) was performed on standardized metabolite data. A heatmap of the top-ranked metabolites was generated using z-score transformation across samples and hierarchical clustering. Metabolite contributions were explored using variable importance in projection (VIP) scores derived from partial least squares discriminant analysis (PLS-DA) as a secondary exploratory feature-ranking approach. VIP scores summarize the relative contribution of each metabolite to the PLS-DA model. In the present study, VIP scores were used only for exploratory feature ranking and not as confirmatory evidence of class separation. Because supervised multivariate methods may yield optimistic results in modest-sized metabolomic datasets, the PLS-DA and VIP findings were interpreted as supportive and hypothesis-generating rather than confirmatory. Pathway-level signals were explored using over-representation analysis (ORA) based on a hypergeometric test within the measured metabolite panel, followed by Benjamini–Hochberg FDR correction.

The sample size was determined pragmatically by the availability of eligible PPCM cases with adequate serum samples during the study period, together with an equal number of controls. Because PPCM is an uncommon condition and the present study was designed as an exploratory case–control metabolomic investigation, no formal a priori sample-size calculation was performed. With 40 participants per group, the study should be regarded as primarily informative for moderate-to-large between-group differences, whereas smaller metabolite effects, pathway-level associations, and multivariable prediction estimates require evaluation in larger independent cohorts. Therefore, the multivariable classification and pathway-level analyses were interpreted as exploratory and hypothesis-generating rather than confirmatory.

## 5. Conclusions

In conclusion, targeted serum organic-acid profiling identified candidate metabolic features associated with peripartum cardiomyopathy, most notably higher homogentisic acid and lower lactic acid levels. These findings suggest a focused organic-acid signal in PPCM, but they should be interpreted as exploratory associations rather than evidence of causal mechanisms or clinically ready biomarkers. Given the modest sample size, single-center case–control design, cross-sectional sampling, and lack of external validation, further prospective studies in larger independent cohorts are required to validate these metabolites, assess their temporal relationship with disease severity and recovery, and determine whether they add value beyond established clinical, echocardiographic, and biomarker-based assessment.

## Figures and Tables

**Table 1:** Baseline characteristics of the study population.

Variable	PPCM (*n* = 40)	Controls (*n* = 40)	*p* Value
**Age, years**	31.8 ± 5.6	30.9 ± 5.1	0.46
**Postpartum interval at sampling, days**	36 (20–63)	34 (19–58)	0.61
**Body mass index, kg/m^2^**	29.8 ± 4.9	28.6 ± 4.4	0.24
**Systolic blood pressure, mmHg**	113 ± 14	110 ± 12	0.29
**Diastolic blood pressure, mmHg**	71 ± 10	69 ± 8	0.33
**Heart rate, beats/min**	96 ± 14	79 ± 10	<0.001
**Left ventricular ejection fraction, %**	31.6 ± 7.8	NA	—
**Left ventricular end-diastolic diameter, mm**	59.2 ± 6.5	NA	—
**White blood cell count, ×10^9^/L**	8.4 ± 2.1	7.8 ± 1.9	0.18
**C-reactive protein, mg/L**	6.2 (3.4–10.7)	4.1 (2.5–6.9)	0.06
**Creatinine, mg/dL**	0.79 ± 0.16	0.74 ± 0.13	0.12
**Estimated glomerular filtration rate, mL/min/1.73 m^2^**	101.5 ± 18.9	107.6 ± 16.3	0.13
**Aspartate aminotransferase, U/L**	27 (21–35)	24 (20–30)	0.17
**Alanine aminotransferase, U/L**	24 (18–33)	21 (16–28)	0.15
**NT-proBNP, pg/mL**	2650 (1480–4820)	NA	—

**Footnote**: Values are presented as mean ± SD or median (IQR), as appropriate. Between-group comparisons were performed using Student’s *t*-test or Mann–Whitney U test according to data distribution. NA: not available.

**Table 2:** Differential metabolites between PPCM and controls (log_2_-normalized abundance).

Metabolite	PPCM (Mean ± SD)	Control (Mean ± SD)	log_2_FC	FC	Hedges g	*p*	q (FDR)
**Homogentisic Acid**	0.74 ± 0.56	0.05 ± 0.53	0.696	1.620	1.269	1.82 × 10^−7^	9.8 × 10^−6^
**Lactic Acid**	−0.44 ± 0.87	0.26 ± 0.77	−0.701	0.615	−0.842	0.000286	0.00773
**2-Oxoadipic acid**	0.34 ± 0.57	0.04 ± 0.50	0.299	1.230	0.555	0.0144	0.259
**Hexanonylglicine**	0.15 ± 0.56	−0.11 ± 0.56	0.260	1.197	0.460	0.0409	0.552
**Succinic Acid**	−0.17 ± 0.67	0.10 ± 0.68	−0.270	0.829	−0.395	0.0782	0.73
**5-Oxoproline**	−0.02 ± 0.68	0.25 ± 0.70	−0.269	0.830	−0.387	0.0843	0.73
**Methyl Malonic Acid**	0.10 ± 0.58	−0.11 ± 0.56	0.212	1.158	0.371	0.0978	0.73
**2 OH 3 Methyl Pentanoic Acid**	0.08 ± 0.70	−0.14 ± 0.67	0.224	1.168	0.324	0.148	0.73
**2-Hydroxyglutaric acid**	−0.14 ± 0.72	0.07 ± 0.69	−0.211	0.864	−0.295	0.187	0.73
**3-Hydroxy-3-methylglutaric acid**	0.20 ± 0.66	0.02 ± 0.61	0.185	1.137	0.289	0.196	0.73
**Fumaricacid**	0.13 ± 0.80	−0.10 ± 0.84	0.229	1.172	0.277	0.215	0.73
**N Acetyltyrosine**	−0.19 ± 0.61	−0.02 ± 0.63	−0.174	0.887	−0.276	0.216	0.73
**2-HydroxyIsocabroic Acid**	0.18 ± 0.67	−0.01 ± 0.78	0.185	1.137	0.252	0.258	0.73
**Sebacic Acid**	−0.03 ± 0.63	0.13 ± 0.59	−0.155	0.898	−0.251	0.261	0.73
**3-Methyl 2-Oxovaleric Acid**	0.13 ± 0.73	−0.06 ± 0.78	0.188	1.139	0.247	0.269	0.73
**Citric Acid**	−0.11 ± 0.64	0.05 ± 0.64	−0.155	0.898	−0.240	0.282	0.73
**2-Hydroxybutyricacid**	0.10 ± 0.67	−0.07 ± 0.73	0.169	1.125	0.239	0.283	0.73
**2-Keto Glutaric Acid**	−0.09 ± 0.65	0.07 ± 0.68	−0.155	0.898	−0.229	0.304	0.73
**Succinylacetone**	0.07 ± 0.65	−0.08 ± 0.65	0.150	1.110	0.229	0.305	0.73
**Subericacid**	−0.11 ± 0.70	0.05 ± 0.65	−0.156	0.897	−0.228	0.305	0.73

Values are presented as mean ± standard deviation (SD). Between-group comparisons were performed using Welch’s *t*-test. Multiple testing was controlled using the Benjamini–Hochberg false discovery rate (FDR). Log_2_ fold change (log_2_FC) was calculated as the difference between the mean log_2_-transformed abundance in the peripartum cardiomyopathy (PPCM) group and that in the control group; positive values indicate higher abundance in PPCM. Fold change (FC) was calculated as 2^(log_2_FC). Effect size is reported as Hedges’ g.

**Table 3:** Classification performance for PPCM vs. controls.

Model	AUC (95% CI)	Decision Threshold	Sensitivity	Specificity
Multivariable logistic regression (5-fold CV)	0.708 (0.590–0.813)	0.211	0.800	0.550
Best single metabolite: Homogentisic acid	0.818 (0.723–0.899)	—	—	—

The multivariable model was evaluated using 5-fold cross-validation. The area under the ROC curve (AUC) is reported with 95% confidence intervals estimated by bootstrap resampling (2000 iterations) of cross-validated predicted probabilities. Sensitivity and specificity correspond to the Youden index–optimal decision threshold. For the best single metabolite model, only AUC (95% CI) is reported.

**Table 4:** Top metabolites ranked by VIP score (PLS-DA, 2 components).

Metabolite	VIP
**Homogentisic Acid**	3.464
**Lactic Acid**	2.520
**2-Oxoadipicacid**	1.829
**Hexanonylglicine**	1.537
**2-OH 3 Methyl Pentanoic Acid**	1.423
**Methyl Malonic Acid**	1.386
**Oxoproline**	1.289
**Succinic Acid**	1.266
**3-OH Butyricacid**	1.129
**Sebacic Acid**	1.017
**2-OH Glutaric Acid**	0.957
**3-OH 3 Methylglutaricacid**	0.925
**4-Methyl 2-Oxovaleric Acid**	0.915
**N-Acetyl Asparticacid**	0.910
**Fumaricacid**	0.910

VIP, variable importance in projection. VIP, variable importance in projection derived from partial least squares discriminant analysis (PLS-DA). Higher VIP values indicate greater contribution to group separation; values > 1 are commonly interpreted as above-average contributors. Because formal PLS-DA validation metrics (e.g., permutation testing, Q^2^, CV-ANOVA) were not generated, VIP findings should be interpreted as exploratory feature-ranking results.

**Table 5:** Pathway enrichment overview (ORA within the measured organic-acid panel).

Pathway	k/K	Enrichment_Ratio	P_Hypergeom	FDR
**Glycolysis/pyruvate metabolism**	1/2	13.500	0.0734	0.66
**Citrate cycle (TCA cycle)**	0/5	0.000	1	1
**Phenylalanine/tyrosine metabolism**	0/7	0.000	1	1
**Propionate metabolism**	0/3	0.000	1	1
**Branched-chain amino acid catabolism**	0/5	0.000	1	1
**Dicarboxylic acid metabolism (omega-oxidation)**	0/5	0.000	1	1

Over-representation analysis (ORA) was performed using a hypergeometric test with the measured metabolite panel as the background universe. Pathway size (K) indicates the number of pathway members present in the dataset; overlap (k) indicates the number of significant metabolites mapping to the pathway. *p* values were adjusted using the Benjamini–Hochberg FDR method. k/K indicates the number of significant metabolites mapping to the pathway divided by the number of measured metabolites in that pathway.

## Data Availability

The data supporting the findings of this study are available in the Supplementary Materials. Additional anonymized data are available from the corresponding author upon reasonable request, subject to privacy and ethical restrictions.

## Supplementary Materials

The following supporting information can be downloaded at: https://www.mdpi.com/article/10.3390/ijms27146451/s1.
